# Parity and diabetes risk among hispanic women from Colombia: cross-sectional evidence

**DOI:** 10.1186/s13098-015-0001-z

**Published:** 2015-02-15

**Authors:** Pablo Cure, Heather J Hoffman, Carlos Cure-Cure

**Affiliations:** Children’s National Health System, 111 Michigan Avenue, NW Washington DC, 20010 USA; The George Washington University, Washington DC, USA; Biomelab, Ltda, Barranquilla, Atlántico Colombia

**Keywords:** Type 2 diabetes, Parity, Hispanic women

## Abstract

**Objective:**

The association between parity and type 2 diabetes has been studied in developed countries and in Singapore and Chinese women but not in Hispanics. Herein we evaluated the association between parity (number of live births) with diabetes in a group of Hispanic postmenopausal women from Colombia.

**Research design and methods:**

Herein we evaluated the association between parity and diabetes in a population of 1,795 women from Colombia. Women were divided in birth categories (0 [referent], 1 or 2, 3–5, 6 or > births). Medical history of diabetes and anthropometric characteristics were recorded. Logistic regressions were performed in order to find the association between parity and diabetes in bivariable and multivariable models after controlling for age, body mass index (BMI), waist hip ratio (WHR) and diabetes family history, among other variables.

**Results:**

In our study, there was an association between parity and diabetes after adjusting for age, BMI and diabetes family history in the multiparous women groups when compared to the women with no births (Referent group) [1–2 births vs. *referent* OR 5.2 (95 CI 1.2–22.9), 3–5 births vs. *referent* OR 5.5 (1.3–23.0) and ≥6 births vs. *referent* OR 7.5 (1.8–31.8), respectively]. The association was maintained in two of the groups in the multivariable analysis [OR 5.0 (1.1–22.9) and 5.3 (1.2–23.5)], for 1 or 2 births and 6 or > births versus 0 births, respectively. Positive diabetes family history and WHR were also associated with an increased risk of diabetes [OR 4.6 (3.0–7.0) and 4.1 (2.0–8.1), respectively].

**Conclusions:**

In postmenopausal Hispanic women, multiparity, as well as a positive family history of diabetes and a high waist-hip ratio were associated with higher diabetes risk.

## Background

During each pregnancy there are known physiological changes, including: insulin resistance, fat accumulation, dyslipidemia, and inflammation [1-6] that could have an impact on the risk of diabetes and other cardio-metabolic disorders later in life.

In multiparous women, the aforementioned physiological changes occurring during each gestation may have lasting effects in the health of the mother which could transcend beyond the gestational period and increase the risk of metabolic syndrome, diabetes and cardiovascular disease later in life [7-12].

Currently there is evidence suggesting an association between childbearing and the risk of type 2 diabetes in women in populations in both developed and developing countries [9-12] however in some cases this association has been lost after controlling for confounding variables such as age and obesity [13]. To our knowledge there are currently no studies evaluating this association in Hispanic women with a high incidence of multi-parity and type 2 diabetes.

In addition pregnancy is associated with significant weight retention (when the woman is not able to return to the original pre-pregnancy weight). This can increase their post pregnancy BMI after each delivery therefore increasing the risk of type 2 diabetes later in life.

Herein we evaluate the association between parity (number of live births) and diabetes in a group of postmenopausal Hispanic women from Colombia. Our main hypothesis was that parity was independently associated with a higher risk of diabetes in our population.

## Methods

This is a cross-sectional study utilizing data collected from medical histories during Health Community Programs at Biomelab Research Center in Barranquilla-Colombia. In this study we tested the association between parity (by number of live births) with type 2 diabetes in 1,795 postmenopausal Hispanic women from Colombia. The study included all post-menopausal women seen at the Health Community Program that had a complete medical history. Women with history of diabetes before their first pregnancy and women with history of hysterectomy were excluded from this analysis. The total number of patients evaluated for this analysis was 3,405 of which 1,795 met the inclusion and exclusion criteria. Data was collected from complete medical histories of women who attended the Health Community Programs between 2004–2007 and included: patient demographics, anthropometric measures (weight [kg], height [cm], Body Mass Index (BMI) [kg/m^2^], waist circumference and hip circumferences [cm]), patient history of CVD, type 2 diabetes and/or other diseases, parity history (total number of live births), breastfeeding history (in months), family history, complete physical exam (including systolic/diastolic blood pressures [mmHg] and heart rate [beats/min]) and smoking history. Women were divided also according to BMI and WHR risk based on the World Health Organization criteria.

Logistic regression analyses were performed to determine whether significant associations between diabetes and parity existed alone, in a bivariable model, and in a multivariable model after controlling for: BMI, age, WHR, smoking history, family history of diabetes and others. For these analyses the Dependent variable was history of diabetes as diagnosed by a doctor and/or based on patient medical history and/or history of any diabetes treatment. The main independent variable studied was number of live births (parity) as a categorical variable divided into the following 4 groups according to previous publications [14]: *nulliparous* (0 deliveries), *one or two deliveries* (1 or 2), *three to five deliveries* (3–5) and *6 or more deliveries* (≥6). In addition a bivariable and multivariable analyses were performed to evaluate the association between family history of diabetes, WHR, smoking history and breastfeeding. Results are presented in percentages, means and standard deviation where appropriate.

The study was approved by the local Institutional Review Board (IRB) in Biomelab, Barranquilla-Colombia and by the The George Washington University and Medical Center, Office of Human Research, IRB board (IRB# 021033).

All patients provided informed consent prior to their participation in the program and for the future utilization of the data obtained.

Data analysis was performed using SAS® version 9.1.

## Results and discussion

A total of 1,795 postmenopausal Hispanic females were included in this analysis. Table 1 displays the mean and standard deviations for continuous variables and frequency distributions for categorical variables.

**Table 1:** **Descriptive statistics (n = 1,795)**

**Variable**	**All women**		**No. of births**			
	**Mean (SD)**		**0 (n = 139)**	**1 or 2 (n = 342)**	**3–5 (n = 758)**	**≥6 (n = 556)**
	**n**	**Mean (SD)**				
Age (yr)	1,795	61.9 (10.0)	61.1 (10.3)	57.7 (9.6)	59.8 (9.3)	67.5 (8.7)
Menarque (yr)	1,795	13.4 (1.8)	13.7 (2.0)	13.2 (1.9)	13.3 (1.8)	13.5 (1.7)
Menopause (yr)	1,795	46.9 (5.8)	46.3 (6.0)	46.3 (5.6)	46.6 (5.8)	47.7 (5.9)
BMI (kg/m^2^)	1,795	26.7 (5.1)	25.6 (5.7)	26.4 (4.6)	27.1 (5.0)	26.7 (5.3)
Waist Circum.(cm)	1,795	86.4 (11.4)	82.9 (12.6)	84.8 (10.9)	86.7 (11.6)	87.9 (11.6)
Hip Circum.(cm)	1,795	101.7(12.2)	98.0 (13.9)	102.0 (11.9)	102.2 (12.1)	101.9 (11.9)
WHR	1,795	0.87 (0.36)	0.90 (0.66)	0.83 (0.08)	0.87 (0.48)	0.87 (0.07)
Systolic BP(mmHg)	1,795	134 (22)	132 (22)	127 (19)	133 (21)	140 (24)
Diastolic BP(mmHg)	1,795	82 (13)	80 (12)	80 (12)	82 (13)	85 (14)
	**n**	**%**	**n (% row)**			
**BMI category**						
Underweight	71	4.2	16 (22.5)	9 (12.7)	25 (35.2)	21 (29.6)
Normal	639	37.6	53 (8.3)	132 (20.7)	262 (41)	192 (30.0)
Overweight	626	36.8	38 (6.1)	114 (18.2)	277 (44.2)	197 (31.5)
Obese	364	21.4	25 (6.9)	62 (17.0)	169 (46.4)	108 (29.7)
**WHR category**						
Low risk	366	22.7	37 (10.1)	89 (24.3)	163 (44.5)	77 (21.1)
Moderate risk	453	28.2	44 (9.7)	96 (21.2)	183 (40.4)	130 (28.7)
High risk	791	49.1	49 (6.2)	123 (15.6)	330 (41.7)	289 (36.5)
**Disease frequency**						
Diabetes	133	7.4	2 (1.4)	21 (6.1)	53 (7.0)	57 (10.3)

BMI = Body Mass Index; WHR = Waist Hip Ratio.

The mean number of live births per women in our population was 4.5 ± 3.0 and the distribution according to parity groups was 8% nulliparous (n = 139), 19% with 1 or 2 deliveries (n = 342), 42% with 3 to 5 deliveries (n = 758) and 31% with 6 or more deliveries (n = 556) (Table 1).

Frequency of diabetes was lower in the nulliparous group when compared to all the other parity groups (1.4% vs. 6.1%, 7.0% and 10.3%, respectively) (Table 1). Women with 6 or more deliveries were older and had higher systolic/diastolic blood pressure compared to the other groups.

In our study there was a dose-dependent increase in the diabetes risk in the bivariable analysis, when comparing each of the parity groups with the referent group [0 births] (Table 2). This association was maintained in the multivariable analysis after adjusting for age, BMI and family history of diabetes, with the odds of diabetes being 5.2 (1.2–22.9), 5.5 (1.3–23.0) and 7.5 (1.8–31.8) times higher, when comparing the referent group with each of the parity groups, respectively. The association remained strong for two of the parity groups, except for the 3–5 deliveries after controlling for more variables including: Age, BMI, Family history of diabetes, WHR, Smoking history, Breastfeeding and Marital Status (Table 2).

**Table 2:** **Odds ratios for the association between parity and diabetes according to the number of births in a bivariable and multivariable analyses**

**Variable**	**No. of subjects affected (%)**	**Odd ratio bivariable analysis**	**Odd ratio adjusted for multivariables** ^**€**^	**Odd ratio adjusted for multivariables** ^**¥**^
		**OR (95% confidence intervals)**		
*Diabetes*	133 (7.4)			
No. of births				
0	2 (1.5)	1.0	1.0	1.0
1 or 2	21 (15.8)	**4.5 (1.0–19.4)**	**5.2 (1.2–22.9)**	**5.0 (1.1–22.9)**
3–5	53 (39.8)	**5.1 (1.2–21.4)**	**5.5 (1.3–23.0)**	4.1 (0.9–17.9)
≥6	57 (42.9)	**7.8 (2.0–32.4)**	**7.5 (1.8–31.8)**	**5.3 (1.2–23.5)**

^€^Adjusted for Age, BMI and Family history of diabetes.

^¥^Adjusted for Age, BMI, Family history of diabetes (Diabetes FHx), WHR, Smoking history, Breastfeeding and Marital Status.

Bolded results = statistically significant p < 0.05.

The overall model containing all the variables was statistically significant (chi^2^ = 106.9, p < 0.0001), and the Hosmer-Lemeshow Goodness of Fit test indicated that the model assumptions were appropriate (Chi2 = 9.2, p = 0.32).

The risk for diabetes according to family history, WHR, smoking history and breastfeeding was evaluated in a bivariable and multivariable adjusted analyses (Table 3). There were strong associations between family history of diabetes and diabetes risk, odd ratio 4.6 (3.0–7.0) and also between WHR risk profile and diabetes risk odd ratio 4.1 (2.0–8.1), in both the bivariable and multivariable models.

**Table 3:** **Relative risk of diabetes according to family Hx, WHR, smoking and breastfeeding in a bivariable and multivariable analyses**

**Variable**	**Odd ratio bivariable analysis**	**Odd ratio adjusted for multivariables** ^**¥**^
	**95% confidence intervals**	
***Diabetes***		
Family Hx of diabetes	**3.8 (2.6**–**5.4)**	**4.6 (3.0**–**7.0)**
Yes (ref) vs. no		
WHR high (ref) vs. low	**4.3 (2.3**–**8.1)**	**4.1 (2.0**–**8.1)**
WHR moderate (ref) vs. low	1.5 (0.7–3.2)	1.6 (0.7–3.5)
Ever smoking	1.0 (0.7–1.6)	1.2 (0.7–1.9)
Yes (ref) vs. no	1.1 (0.8–1.6)	0.9 (0.6–1.4)
Breastfeeding (months)		
6 or more (ref) vs. 0–6		

^¥^Adjusted for Age, BMI, Family history of diabetes (Diabetes FHx), WHR risk, Smoking history, Breastfeeding history and Marital Status Bolded results = statistically significant p < 0.05.

Ref = Referent Group.

## Conclusions

This is the first study investigating the association between parity and diabetes risk exclusively in a Hispanic population. In our study, we found that multiparous women had a 5–7 fold increased risk of diabetes when compared to their nulliparous counterparts.

In a recent large, prospective cohort of Singaporean Chinese women NT Mueller et al. [11] found strong and graded positive association between parity and type 2 diabetes , after adjusting for various demographic, lifestyle, reproductive health factors and BMI.

The most recent study by Y Tian et al. [12] found that fasting plasma glucose level and the prevalence of diabetes were associated with the number of live births suggesting that higher parity is a risk factor for diabetes in their population of Chinese women. This contrasts with early studies showing slight association between parity and diabetes that was then lost after controlling for confounding variables such as age and weight [13].

In addition, prospective studies in other populations have found an increase in the risk of metabolic syndrome in multiparous women compared to nulliparous [7,8] with an 11% increase in the risk of metabolic syndrome with each delivery [7]. Furthermore, the CARDIA study showed a direct association between childbearing and incidence of metabolic syndrome among women with gestational diabetes mellitus but not among women without it [8]. Other studies are consistent with our findings, including Kritz-Silverstein et al. [9] who examined the relationship between parity and prevalence of diabetes in a population-based study of 1,186 women aged 41–92 years, and found that the risk of diabetes increased slightly with the number of live births independent of age, BMI and family history of diabetes. Other prospective studies after adjustment for sociodemo-graphic factors and body fat only observed a greater risk among gran multiparous women [10].

Our study also found that women in the *Higher Risk* category for WHR had 4.3 fold risk of diabetes when compared to women in the the *Low Risk* WHR. These results are in agreement with other studies showing association between this important anthropometric measure and diabetes risk [15,16]. Furthermore, we found that family history of diabetes (FHx) was significantly associated (OR 4.6) with diabetes.

It is worth noted, that in our population, the prevalence of type 2 diabetes was 7.4% which is slightly lower of that reported in other studies, including the Behavioral Risk Factors Surveillance System (BRFSS), which reported an age-adjusted self-reported prevalence of diabetes among Hispanics of 9.8% [17] and a study by Lao et al. that showed a 9.7% prevalence of diabetes in a large cohort of Chinese women age 50–93 years old [7]. The lower prevalence in our studied population could be explained by under-diagnosis of diabetes. In an additional analysis of our data including only 1,098 from our population that had fasting blood glucose results, we found that the prevalence of diabetes by history in this population was 8.7% and, in the same population, the prevalence of diabetes by fasting blood glucose results was 11.9%, suggesting that the percentage of undiagnosed diabetes in our population was about 3.2%, similar to the 3% prevalence of undiagnosed diabetes reported by *Cowie et al.* for Mexican-Americans [18].

The fact that populations in Latin American countries are showing a higher tendency towards developing metabolic diseases (ie. type 2 diabetes) and cardiovascular disease has been recognized [18,19]. Furthermore, the US National Health and Nutrition Examination Survey (NHANES) showed higher rates of overweight and obesity in minority groups when compared to non-Hispanic whites [20]. These higher risk for metabolic disorders coupled with a “high” parity rate may both be contributing factors for the markedly increased risk of type 2 diabetes observed in our population.

Prospective studies, especially in populations with similar characteristics, should be carried-out in order to more accurately answer our hypothesis. Still our results can be an early and reliable source of information that could help designing disease prevention programs while gaining 10 years in the process and while we wait for the results of prospective studies [21].

One of the recommendations, based on this study, is to suggest additional laboratory work-up as part of the routine check-up that may include fasting glucose and an oral glucose tolerance test (when recommended) in postmenopausal multiparous and gran multiparous women, especially those of Hispanic origin. Early prevention programs in postmenopausal women need to include family history of diabetes, increased WHR and multi-parity as important risk factors for diabetes in this population. In addition, well designed programs to prevent excessive weight gain during each delivery may help diminish the health impact of parity, later on in life.

The implications of our results in other Hispanic populations, including Hispanics living in developed countries, such as in the United States, are unknown. Environmental factors, such as high-caloric foods, lack of exercise and other lifestyle factors differ between Hispanics living in their countries of origin and Hispanics in the US. These differences make the results in our populations difficult to extrapolate to the US Hispanic population, but our recommendations of including multiparity in the risk profile for postmenopausal women and developing prevention programs tailored to this population may also be applicable to Hispanic women in the US.

As any cross-sectional study, our study has several limitations. One of them is the lack of information about the onset of diabetes according to the time of each birth which could be a factor for patients that developed the disease before their last birth which may overestimate the association between diabetes and parity in the higher parity groups. Another limitation is the lack of information about gestational diabetes or the history of macrosomia in any of the newborns. Finally, weight gain after each delivery was not recorded and this could be an important factor in the development of diabetes in these populations.

In conclusion, in our study we found a strong association between parity and diabetes in postmenopausal Hispanic women as well as an association between positive family history of diabetes and high WHR with diabetes in the same population.

## Abbreviations

BMI: Body mass index
BRFSS: Behavioral risk factors surveillance system
CVD: Cardiovascular disease
IRB: Institutional Review Board
NHANES: The US National Health and Nutrition Examination Survey
WHR: Waist Hip ratio
